# Complete Genome Sequence of Genotype Ib Newcastle Disease Virus Isolated from a Mallard (*Anas platyrhynchos*) in Russia

**DOI:** 10.1128/genomeA.01414-15

**Published:** 2015-12-03

**Authors:** Kseniya S. Yurchenko, Ivan A. Sobolev, Alexandra V. Glushchenko, Alexander M. Shestopalov

**Affiliations:** FSBSI Research Institute of Experimental and Clinical Medicine, Novosibirsk, Russia

## Abstract

We report here the complete genome sequence of a Newcastle disease virus isolate, NDV/Yakutiya/mallard/852/2011, isolated from a mallard in Russia. On the basis of phylogenetic analysis, this strain was clustered into class II genotype Ib.

## GENOME ANNOUNCEMENT

Newcastle disease (ND) is a highly contagious viral disease caused by virulent avian paramyxovirus serotype 1 (APMV-1), also known as Newcastle disease virus (NDV) (1). There is also an antigenic variant of NDV, which has appeared in pigeons (2). The causative agent, which has a single-stranded negative-sense RNA genome, belongs to the genus *Avulavirus* (3).

NDV pathotypes are based on clinical signs in infected chickens: velogenic, mesogenic (intermediate), and lentogenic with mild infection (1, 4). However, the clinical signs of viral infections by different pathotypes can vary with the bird species. Wild ducks are regarded natural reservoirs of avirulent NDV strains and in most cases display few or no symptoms consistent with ND (5, 6). Nonetheless, several strains might be more virulent in chickens after passaging from infected to healthy birds (7, 8).

In the present study, we report the complete genome sequence of NDV/Yakutiya/mallard/852/2011 strain, initially isolated from a clinical healthy wild mallard (*Anas platyrhynchos*) in the Russian Far East (Republic of Sakha [Yakutiya]) in fall of 2011.

Nine-days-old embryonating chicken eggs were used for viral propagation. Standard pathogenicity assays were performed, including determinations of the mean death time (MDT) and intracerebral pathogenicity index (ICPI) (9). NDV/Yakutiya/mallard/852/2011 is classified as mesogenic in chickens, with an ICPI of 1.04 and MDT of 116 h.

Viral RNA was extracted from allantoic fluid using TRIzol LS (Invitrogen, USA). cDNA was synthesized using random hexamer primer with RevertAid Premium (Thermo Scientific, USA). The second DNA chain was synthesized using the NEBNext mRNA second-strand synthesis module (NEB, USA), in accordance with the manufacturer’s instructions. DNA libraries were made using TruSeq DNA sample prep kits version 2 (Illumina, USA), as specified by the manufacturer. The DNA sequence of the libraries was made using MiSeq (Illumina, USA) with the reagent kits version 2 (300 paired-end [PE]), according to the manufacturer’s instructions. Bioinformatic analysis was conducted using the CLC Genomics Workbench 5.5 software (CLC bio, USA).

The complete genome of NDV/Yakutiya/mallard/852/2011 has been sequenced and found to be 15,186 nucleotides (nt) in length. The fusion (F) protein cleavage site is one of the main determinants of NDV pathogenicity (1), for which sequence analysis shows that NDV/Yakutiya/mallard/852/2011 contains the typical avirulent type sequence 112G-K-Q-G-R116, with leucine at residue 117. A phylogenetic tree classified the strain into class II genotype Ib. Viruses of this genotype are mainly of low virulence and are often detected in wild and domestic waterfowl. It is supposed that waterbird isolates from the Far East of separate sublineage Ib are the possible transmission between the wild and domestic waterfowl (10).

Phylogenetic analysis of the F gene sequence showed a high similarity of NDV/Yakutiya/mallard/852/2011 with strains from South Korea (GenBank accession no. JX401405), Japan (GenBank accession no. KC503411) (11), China (GenBank accession no. KM885166) (12), and other closely related NDV isolates from the Russian Far East (GenBank accession no. KC503482) (11), North America (11, 13) and North Europe (Sweden) (14).

Thus, we first isolated genotype Ib NDV in the Republic of Sakha (Yakutiya), where there is no poultry in the region; therefore, the most likely way virus of this sublineage could appear in the present territory is by viral transmission from migratory birds.

### Nucleotide sequence accession number.

The complete genome sequence of NDV/Yakutiya/mallard/852/2011 has been deposited in GenBank under the accession no. KJ920203.
